# Supplementary material for: The impact of functional medicine on patient-reported outcomes in inflammatory arthritis: A retrospective study
Source: PLoS One. 2020 Oct 8;15(10):e0240416. doi: 10.1371/journal.pone.0240416 (PMC7544031; doi:10.1371/journal.pone.0240416)
Supplement: Attachment — Submitted filename: Response to Reviewers.docx [file pone.0240416.s001.docx]

PONE-D-20-14349
The Impact of Functional Medicine on Patient-Reported Outcomes in Inflammatory Arthritis: A Retrospective Study
PLOS ONE


This a relatively small study, The research hypothesis, sample size calculation, power analyses and the methodology used in the study need to be re-evaluated.

1. 1. Please ensure that your manuscript meets PLOS ONE's style requirements, including those for file naming. The PLOS ONE style templates can be found at

[https://journals.plos.org/plosone/s/file?id=wjVg/PLOSOne_formatting_sample_main_body.pdf](https://protect-us.mimecast.com/s/ICYsCkRKpWCO5LN7lCJESKy?domain=journals.plos.org) and

[https://journals.plos.org/plosone/s/file?id=ba62/PLOSOne_formatting_sample_title_authors_affiliations.pdf](https://protect-us.mimecast.com/s/Ujf_ClYLqWS2X97yGcYDpXn?domain=journals.plos.org)

**We appreciate your feedback regarding the formatting. We have corrected and reformatted the manuscript to meet PLOS ONE’s style requirements**

2. Thank you for stating the following in the Competing Interests section:

'I have read the journal's policy and the authors of this manuscript have the following competing interests:
Nicole Droz, MD- none
Yuxuan Jin- none
Mark Hyman, MD- * Ownership of stocks or shares:  Mutual funds and investments that may include medically or food related companies managed by investment broker,, multiple private companies including Thrive Market, Hu Kitchen, Walden Meats,  WTR MLN WTR, Parsley Health, Bulletproof, Good Money

* Paid employment or consultancy: Cleveland Clinic and owner of The UltraWellness Center, Hyman Digital, Hyman Enterprises (published 16 books on health and nutrition), Vitamin Portfolio, Farmacy, The Doctor’s Farmacy podcast

* Board membership: Institute for Functional Medicine, Environmental Working Group, Center for Mind Body Medicine

Michelle Beidelschies, PhD: reported receiving personal fees from Cleveland HeartLab, Inc. outside the submitted work. In addition, Dr. Beidelschies had a patent (No. 20110269150) issued for compositions and methods for predicting cardiovascular events.

M. Elaine Husni, MD MPH: Dr. Husni is a consultant (with honoraria) from AbbVie, Janssen, Sanofi Genzyme/Regeneron, UCB, Novartis, and Lilly (less than $10,000 each) and is a coinventor on a patent for a psoriatic arthritis questionnaire PASE (Psoriatic Arthritis Screening Evaluation), for which she receives royalties.

Patrick Hanaway, MD: Dr Hanaway reported serving as paid educational consultant and teaching for the Institute for Functional Medicine.'

We note that you have a patent relating to material pertinent to this article.

Please provide an amended statement of Competing Interests to declare this patent (with details including name and number), along with any other relevant declarations relating to employment, consultancy, patents, products in development or modified products etc.

**We appreciate your diligence in evaluating any potential competing interests. We agree that it is important to fully disclose any potential conflicts of interest related to submitted research. Dr. Beidelschies has a patent issued for compositions and methods for predicting cardiovascular events. This was not used in this study and was outside the scope of this submitted manuscript. The patent number was included in the competing interest section.**

**In the previously submitted manuscript, it was stated that Dr. Husni was a “coinventor on a patent for a psoriatic arthritis questionnaire PASE.” The PASE questionnaire is copyrighted but not patented. This error has been changed in the manuscript and cover letter.**

**For clarification, the PASE questionnaire is a patient self-administered 15 item questionnaire used as an aid to diagnose psoriatic arthritis in patients who have psoriasis. Patients in our study already had psoriatic arthritis confirmed by a board certified rheumatologist and were not given this questionnaire. This does not alter our adherence to PLOS ONE policies.**

**The manuscript and cover letter were updated to reflect these changes:**

“Disclosures: Nicole Droz, MD- none

Yuxuan Jin- none

Mark Hyman, MD- * Ownership of stocks or shares: Mutual funds and investments that may include medically or food related companies managed by investment broker,, multiple private companies including Thrive Market, Hu Kitchen, Walden Meats, WTR MLN WTR, Parsley Health, Bulletproof, Good Money

* Paid employment or consultancy: Cleveland Clinic and owner of The UltraWellness Center, Hyman Digital, Hyman Enterprises (published 16 books on health and nutrition), Vitamin Portfolio, Farmacy, The Doctor’s Farmacy podcast

* Board membership: Institute for Functional Medicine, Environmental Working Group, Center for Mind Body Medicine

Michelle Beidelschies, PhD: reported receiving personal fees from Cleveland HeartLab, Inc. outside the submitted work. In addition, Dr. Beidelschies had a patent (No. 20110269150) issued **for compositions and methods for predicting cardiovascular events.** .

M. Elaine Husni, MD MPH: Dr. Husni is a consultant (with honoraria) from AbbVie, Janssen, Sanofi Genzyme/Regeneron, UCB, Novartis, and Lilly (less than $10,000 each) and is **a coinventor on a psoriatic arthritis questionnaire PASE (Psoriatic Arthritis Screening Evaluation) which is copyrighted, for which she receives royalties.**

Patrick Hanaway, MD: Dr Hanaway reported serving as paid educational consultant and teaching for the Institute for Functional Medicine.

**This does not alter our adherence to  PLOS ONE policies on sharing data and materials.”**

Please confirm that this does not alter your adherence to all PLOS ONE policies on sharing data and materials, as detailed online in our guide for authors [https://protect-us.mimecast.com/s/1r_nCmZ6rWHjW02ZXSNPNsM?domain=journals.plos.org](about:blank) by including the following statement: "This does not alter our adherence to  PLOS ONE policies on sharing data and materials.” If there are restrictions on sharing of data and/or materials, please state these. Please note that we cannot proceed with consideration of your article until this information has been declared.

This information should be included in your cover letter; we will change the online submission form on your behalf.

Please know it is PLOS ONE policy for corresponding authors to declare, on behalf of all authors, all potential competing interests for the purposes of transparency. PLOS defines a competing interest as anything that interferes with, or could reasonably be perceived as interfering with, the full and objective presentation, peer review, editorial decision-making, or publication of research or non-research articles submitted to one of the journals. Competing interests can be financial or non-financial, professional, or personal. Competing interests can arise in relationship to an organization or another person. Please follow this link to our website for more details on competing interests: [https://protect-us.mimecast.com/s/1r_nCmZ6rWHjW02ZXSNPNsM?domain=journals.plos.org](about:blank)

4. Please justify the sample size included in this study, i.e. with reference to any sample size calculations performed or by citing previous studies.

Please refer to our statistical reporting guidelines for assistance ([https://journals.plos.org/plosone/s/submission-guidelines.#loc-statistical-reporting](https://protect-us.mimecast.com/s/BeU5Cn5XvWu7my5zwH0UyO2?domain=journals.plos.org)).

**We appreciate your concern about justifying sample size. When initially creating the study design we also had this concern. Prior to enrolling patients, we had our statistician perform an analysis based on prior studies to address this question to ensure adequate enrollment. This was added to the paper to justify our sample size.**

**Statistical analysis was performed by our statistician, Yuxuan Jin, who is a co-author on the paper.**

**The formal sample size calculation is now included in the statistical analysis section. According to the calculation, a total of 40 patients was recommended. Patients were evaluated over the course of a 15 month time span for inclusion into the study. All patients who met inclusion criteria were included in the final analysis. As a result, our sample size of 109 (54 in FM, 55 in Standard of Care) should have enough power for our analysis.**

**The following was added to the statistical analysis section of the paper:**

“Patient-Reported Outcomes

The outcomes of the study were 12 weeks to baseline changes in pain and PROMIS global physical and mental health scores. PROMIS is a set of validated patient-centered measures, developed by the National Institute of Health, that evaluate and monitor physical, mental and social health. This patient self-administered questionnaire was created to be relevant across all conditions for assessment of functionality, symptoms, behaviors and feelings[10]. In particular, this study utilized the PROMIS Scale v1·2- Global Health measure at baseline and at 12 weeks. PROMIS is a precise and reliable way to measure domains of physical, mental and social health that are critical to PtGA.6 Pain scores were collected using visual analog scale ranging from 0-10 at baseline and 12 weeks.

**Statistical analysis**

**Sample size was calculated using a two-sample t-test with a 5-point differential in mean PROMIS score changes, and a standard deviation of 5.15. This provided 80% power at the 5% significant level. The sample size required to achieve adequate power for PROMIS outcomes was 36 patients total, assuming 18 patients in each group. To account for a 10% attrition rate, it was recommended that at least 20 patients be assigned to each group (40 patients, total).”**

Additional Editor Comments:

Add the limitations of study in the abstract and discussion.

**Thank you for your feedback, limitations were added in the abstract:**

**“Limitations: Retrospective design, baseline difference in patient reported outcomes”**

**We also expanded upon additional limitations in the discussion:**

“Our study had several limitations. First, was the retrospective nature of our study. Because patients were not randomized, the patients who participated in the functional medicine program may have an inherent bias towards not using chronic disease modifying therapy. This was illustrated by the differences in baseline treatment regimens. More patients in the functional medicine group were on no RA or PsA disease modifying therapy as compared to patients in the standard of care group. RAPID3 scores were similar between groups indicating that this difference in treatment was not reflective of differences in disease activity. Without prospectively randomizing patients, we were unable to achieve homogeneity regarding treatment or certain lifestyle behaviors between groups. **For instance, a higher number of patients in the standard of care group were smokers as compared to patients in the functional medicine group. Although this was not statistically significant, it is well known that cigarette smoking increases rheumatoid arthritis disease severity [16].** It is possible that the statistically significant differences in PROMIS physical health and pain scores favoring the functional medicine group is reflective of milder disease or shorter duration of disease as opposed to those patients receiving standard of care in rheumatology alone.”

Calculate sample size and perform power analyses.

The paper needs to be reviewed by a statistician to address whether the methodology used can address the research objectives

**Thank you for your comments, as discussed above in #4, we have added our sample size calculation.**


[Note: HTML markup is below. Please do not edit.]

Reviewers' comments:

Reviewer's Responses to Questions

**Comments to the Author**

1. Is the manuscript technically sound, and do the data support the conclusions?

The manuscript must describe a technically sound piece of scientific research with data that supports the conclusions. Experiments must have been conducted rigorously, with appropriate controls, replication, and sample sizes. The conclusions must be drawn appropriately based on the data presented.

Reviewer #1: Yes

Reviewer #2: Partly

Reviewer #3: Yes

2. Has the statistical analysis been performed appropriately and rigorously?

Reviewer #1: I Don't Know

Reviewer #2: No

Reviewer #3: Yes

3. Have the authors made all data underlying the findings in their manuscript fully available?

The [PLOS Data policy](https://protect-us.mimecast.com/s/zQmWCo2KwWUrvo4M9h2ri3S?domain=plosone.org) requires authors to make all data underlying the findings described in their manuscript fully available without restriction, with rare exception (please refer to the Data Availability Statement in the manuscript PDF file). The data should be provided as part of the manuscript or its supporting information, or deposited to a public repository. For example, in addition to summary statistics, the data points behind means, medians and variance measures should be available. If there are restrictions on publicly sharing data—e.g. participant privacy or use of data from a third party—those must be specified.

Reviewer #1: Yes

Reviewer #2: Yes

Reviewer #3: Yes

4. Is the manuscript presented in an intelligible fashion and written in standard English?

PLOS ONE does not copyedit accepted manuscripts, so the language in submitted articles must be clear, correct, and unambiguous. Any typographical or grammatical errors should be corrected at revision, so please note any specific errors here.

Reviewer #1: Yes

Reviewer #2: Yes

Reviewer #3: Yes

5. Review Comments to the Author

Please use the space provided to explain your answers to the questions above. You may also include additional comments for the author, including concerns about dual publication, research ethics, or publication ethics. (Please upload your review as an attachment if it exceeds 20,000 characters)

Reviewer #1: Grammatical errors (per Line number):
1. L30: “Despite, treatment” –remove coma

**The comma was removed**

2. L30:“arthritis ,” -- remove empty space

**The** **empty space was removed**

3. L34: “patient reported” –hyphenate

**This was changed to patient-reported**

4. L39: “Multivariable”—should probably be the more common MULTIVARIATE

**Although the terms “multivariable” and “multivariate” are sometimes used inter-changeably, “multivariable” would be the correct term to use here. In our study, we have built models with multiple independent variables (x’s). “Multivariate”, on the other hand, should refer to a model where multiple outcome variables were included.**

5. L40: be consistent with either “patients reported” or patient-reported

**This was changed to patient-reported**

6. L49: “patient’s” – should be patient or patients’

**This was changed to patients’**

7. L61: “outcomes, however” –use semicolon or start new sentence

**A semicolon was added**

8. L65: “physician assessed” –hyphenate

**This was** **hyphenated to physician-assessed**

9. L115: “regiment” is a military unit. regimen is a prescribed course of medical treatment

**This was changed to regimen**

10. L126: “ex.” –nonstandard abbreviation, should be e.g.,

**This was changed to e.g.**

11. L144: “factorwere” – separate conjoined words

**These words separated to “factor were”**

Research limitations/concerns:
1. L221: Limitations should also include mention that more patients in the standard treatment group were smokers, as smoking = worse prognosis.

**We appreciate the close attention to lifestyle habits and agree that smoking portends worse prognosis in both psoriatic and rheumatoid arthritis. Although patients who smoked were numerically higher in the standard of care group as opposed to the functional medicine group, this did not meet statistical significance. We added additional discussion regarding this as a potential limitation to the manuscript as detailed below:**

“Our study had several limitations. First, was the retrospective nature of our study. Because patients were not randomized, the patients who participated in the functional medicine program may have an inherent bias towards not using chronic disease modifying therapy. This was illustrated by the differences in baseline treatment regimens. More patients in the functional medicine group were on no RA or PsA disease modifying therapy as compared to patients in the standard of care group. RAPID3 scores were similar between groups indicating that this difference in treatment was not reflective of differences in disease activity. Without prospectively randomizing patients, we were unable to achieve homogeneity regarding treatment or certain lifestyle behaviors between groups. **For instance, a higher number of patients in the standard of care group were smokers as compared to patients in the functional medicine group. Although this was not statistically significant, it is well known that cigarette smoking increases rheumatoid arthritis disease severity [16].** It is possible that the statistically significant differences in PROMIS physical health and pain scores favoring the functional medicine group is reflective of milder disease or shorter duration of disease as opposed to those patients receiving standard of care in rheumatology alone.”

2. Needs contextualization: Pain dropped from 3.5 to 3.1 in the treatment group and 5.2 to 5.0 in the standard care group. Given that these are derived from a 0-10 scale, I doubt that these are of clinical importance.

**Thank you for shedding light on this important topic. We agree that it is necessary to understand both the statistical significance and the clinical significance of results when interpreting studies. As a whole, there was a statistically significant reduction in pain in the functional medicine group as compared to the standard of care group. Because of the baseline differences in pain scores, a multivariable analysis was performed and demonstrated the functional medicine group had a reduction in pain score of -0.92 compared to the standard of care group. The minimal clinically significant difference in pain scores in patients with rheumatoid arthritis has previously been reported as a reduction of -0.5 to -1.1 in observational studies. Therefore, the reductions in the pain score in the functional medicine group are both clinically and statistically significant. This was added to the discussion in the manuscript to offer contextualization:**

**“**In this retrospective study, the adjunctive treatment with a 12-week functional medicine program improved PROMIS physical and mental health scores as well as pain scores as compared to the standard of care. These results are important new knowledge for healthcare professionals when discussing adjunctive care related to nutrition and lifestyle habits in RA and PsA patients. Since prior research has been largely limited to both anecdotal and observational analyses for these alternative care models, these results support the use of adjunctive care with a functional medicine approach to improve patient reported outcomes in RA and PsA patients.

**Due to the baseline characteristic differences and wide distribution of responses, a multivariable analysis was performed and demonstrated a statistically significant improvement in pain scores of -0.92 in the functional medicine group as compared to the standard of care group. Previous observational studies have reported the minimal clinically significant difference in pain scores in patients with rheumatoid arthritis as a reduction of pain on a 10 point VAS scale of -0.5 to -1.1 [12]. Therefore, although the reductions in pain score in the functional medicine group were small, they were both clinically and statistically significant.**

**Similarly, PROMIS physical health T scores were greater by 2.84 points in the functional medicine group as compared to standard of care when controlling for baseline differences. Previous studies have suggested that the minimal clinically significant difference in PROMIS physical health scores range from 1.9 to 2.2 indicating this was also both statistically and clinically significant [13].”**

3. Needs clarification: “318 patients were screened for inclusion into the functional medicine group. … The main reasons for exclusion from the study were … escalation of medical therapy for PsA or RA.” If the treatment did not work and drugs were escalated, then the patients were excluded from the final calculations, thereby biasing for treatment efficacy? 264 eliminated from 318 = 83% eliminated.

**We appreciate the reviewers concern for the potential for treatment bias. Given the retrospective nature of this study, the majority of patients were excluded because of incomplete data or inadequate follow up during the studied time frame. We do not feel this resulted in any bias of the results.**

4. Zero mention of cost, therefore impossible to consider cost-effectiveness. However, given the notoriously high cost of functional medicine care, this is an important omission.

**We agree cost is an important aspect in considering options for patient care. Functional Medicine care (as delivered at Cleveland Clinic) is not fee-for-service, but rather insurance based. Insurance covers medical visits, most laboratory testing and visits with allied health professionals (including dieticians). There are out-of-pocket costs associated with targeted dietary supplementation, but not the medical visits. However, the aim of the current study was not to evaluate the cost effectiveness of a functional medicine model vs. standard of care treatment. Current studies through the Center for Functional Medicine are evaluating this important economic question.**

Reviewer #2: The authors report the benefits of functional medicine, an adjuvant form of therapy, in patients with chronic inflammatory arthritis, specifically rheumatoid arthritis and psoriatic arthritis. The study is original and interesting and opens a new avenue for an adjuvant approach to this type of disease.
Before considering publication of this manuscript, this reviewer has the following questions and suggestions.
1. Please define the abbreviation PROMIS in the abstract

**PROMIS was defined as Patient Reported Outcomes Measurement Information System**

2. In the introduction, delve deeper into the concept of functional medicine and what the benefits of this form of treatment are based on. For example, substantiate how this model can influence the immunoinflammatory pathways of both diseases, or if there is any study that has analyzed this intervention on intestinal dysbiosis found in both diseases.

**We agree that further studies into the mechanistic pathways of a functional medicine approach. At this time, there are limited studies looking at mechanistic changes in patients with inflammatory arthritis but early literature does support microbiome effects on inflammatory disease. The aim of this study was not to evaluate the influence of a functional medicine approach on the immunoinflammatory pathways of disease.**

**Hannah Jethwa, Sonya Abraham, The evidence for microbiome manipulation in inflammatory arthritis, Rheumatology, Volume 56, Issue 9, September 2017, Pages 1452–1460**

**Matsumoto Y, Sugioka Y, Tada M, et al. Monounsaturated fatty acids might be key factors in the Mediterranean diet that suppress rheumatoid arthritis disease activity: The TOMORROW study. *Clin Nutr*. 2018;37(2):675-680.**

**Maeda Y, Takeda K. Role of Gut Microbiota in Rheumatoid Arthritis. *J Clin Med*. 2017;6(6):60. Published 2017 Jun 9.**

3. Although the authors provide a good methodological description, fundamental information is missing. For example, we know nothing of the pharmacological regimens of both groups; Nor are we told if both groups were adequately matched for these treatments, including their duration; nothing is said about what the inclusion criteria are for subjecting patients to functional medicine. In sum, the authors must provide detailed information on both groups, including information on both types of arthritis patients.

**We understand the concerns of the reviewer regarding differences in pharmacologic regimens between groups. We have now included treatment regimens to table 1:**

| **Table 1. Descriptive statistics on patient reported outcomes at baseline, demographic and clinical characteristics** | | | | | | | | | | |
| --- | --- | --- | --- | --- | --- | --- | --- | --- | --- | --- |
|  | **Overall (N=109)** | |  | **Functional Medicine (N=54)** | |  | **Standard of Care  (N=55)** | |  |  |
| **Factor** | **N** | **Statistics** |  | **n** | **Statistics** |  | **n** | **Statistics** |  | **p-value** |
| Global physical health T-score baseline | 109 | 41.4±7.9 |  | 54 | 43.2±6.6 |  | 55 | 39.7±8.7 |  | ***0.022^a^*** |
| Global mental health T-score baseline | 109 | 45.6±8.3 |  | 54 | 45.8±8.4 |  | 55 | 45.3±8.3 |  | 0.73^a^ |
| Pain score baseline | 108 | 4.4±2.5 |  | 54 | 3.5±1.9 |  | 54 | 5.2±2.7 |  | ***<0.001^a^*** |
| RAPID3 baseline | 51 | 3.9±2.0 |  | 6 | 3.2±1.02 |  | 45 | 4.0±2.1 |  | 0.36^a^ |
| Age | 109 | 52.9±11.3 |  | 54 | 51.0±10.6 |  | 55 | 54.8±11.8 |  | 0.078^a^ |
| Sex | 109 |  |  | 54 |  |  | 55 |  |  | 0.075^c^ |
| . male |  | 35(32.1) |  |  | 13(24.1) |  |  | 22(40.0) |  |  |
| . female |  | 74(67.9) |  |  | 41(75.9) |  |  | 33(60.0) |  |  |
| Smoking | 108 |  |  | 53 |  |  | 55 |  |  | 0.16^c^ |
| . Never |  | 62(57.4) |  |  | 34(64.2) |  |  | 28(50.9) |  |  |
| . Former or Current |  | 46(42.6) |  |  | 19(35.8) |  |  | 27(49.1) |  |  |
| CCP | 109 |  |  | 54 |  |  | 55 |  |  | 0.67^c^ |
| . Positive |  | 43(39.4) |  |  | 23(42.6) |  |  | 20(36.4) |  |  |
| . Negative |  | 40(36.7) |  |  | 20(37.0) |  |  | 20(36.4) |  |  |
| . Unknown |  | 26(23.9) |  |  | 11(20.4) |  |  | 15(27.3) |  |  |
|  |  |  |  |  |  |  |  |  |  |  |
| RF | 109 |  |  | 54 |  |  | 55 |  |  | 0.12^c^ |
| . Positive |  | 37(33.9) |  |  | 22(40.7) |  |  | 15(27.3) |  |  |
| . Negative |  | 50(45.9) |  |  | 25(46.3) |  |  | 25(45.5) |  |  |
| . Unknown |  | 22(20.2) |  |  | 7(13.0) |  |  | 15(27.3) |  |  |
| Treatment at baseline | 108 |  |  | 53 |  |  | 55 |  |  | ***<0.001^C^*** |
| . Conventional DMARD therapy |  | 29(26.9) |  |  | 12(22.6) |  |  | 17(30.9) |  |  |
| . Biologic DMARD therapy |  | 66(61.1) |  |  | 28(52.8) |  |  | 38(69.1) |  |  |
| . None |  | 13(12.0) |  |  | 13(24.5) |  |  | 0(0.0) |  |  |
| Statistics presented as Mean ± SD or N (column %). p-values: a=t-test, c=Pearson's chi-square test.  CCP= anti-cyclic citrullinated peptide; RF= rheumatoid factor; DMARD= Disease modifying anti-rheumatic drug | | | | | | | | | | |

**We also included further information in the results:**

“318 patients seen in the functional medicine department were initially identified as having RA or PsA by ICD 9/10 code. Of these patients, 54 had documentation of their diagnosis by a board certified rheumatologist and had complete data over the study time frame and were included into the study. The main reasons for exclusion from the study were: incomplete data, inadequate follow up within the first 12 weeks after starting the functional medicine program, inadequate documentation of rheumatologic diagnosis or confounding by lack of stable doses of medications to treat inflammatory arthritis. In the standard of care group, 54 patients seen in the rheumatology department alone without any functional medicine intervention were identified for inclusion into the study. Baseline characteristics were similar between both groups with the exception of PROMIS global physical health and pain scores which were significantly different in the functional medicine group as compared to the standard of care group (PROMIS global physical health score 43·2 ± 6·6 in the functional medicine group as compared to 39·7 ± 8·7 in the standard of care group and pain scores of 3·5 ± 1·9 and 5·2 ± 2·7 respectively) (Table 1). **There was also a statistically significant difference among treatment regimens. This was likely due to a high number of patients in the functional medicine group who were risk averse to using chronic disease modifying therapy. Interestingly disease activity scores as measured by RAPID3, a validated disease assessment instrument, were similar despite these treatment differences.”**

**And discussion sections:**

“Our study had several limitations. First, was the retrospective nature of our study. Because patients were not randomized, the patients who participated in the functional medicine program may have an inherent bias towards not using chronic disease modifying therapy. **This was illustrated by the differences in baseline treatment regimens. More patients in the functional medicine group were on no RA or PsA disease modifying therapy as compared to patients in the standard of care group. RAPID3 scores were similar between groups indicating that this difference in treatment was not reflective of differences in disease activity.** Without prospectively randomizing patients, we were unable to achieve homogeneity regarding treatment or certain lifestyle behaviors between groups. For instance, a higher number of patients in the standard of care group were smokers as compared to patients in the functional medicine group. Although this was not statistically significant, it is well known that cigarette smoking increases rheumatoid arthritis disease severity [16]. It is possible that the statistically significant differences in PROMIS physical health and pain scores favoring the functional medicine group is reflective of milder disease or shorter duration of disease as opposed to those patients receiving standard of care in rheumatology alone. However, using a multivariate analysis, we were able to account for these differences seen between groups and were able to demonstrate a statistically significant improvement in all primary outcomes in patients treated with an adjunctive functional medicine program. “

**There was a statistically significant difference between treatments between group. This was likely due to a high number of patients in the functional medicine group who were risk averse to chronic disease modifying therapy. RAPID3 scores were similar between groups indicating that differences in treatment regimens were not due to differences in disease activity.**

**In this retrospective observational study, patients were not assigned into any specific RA/PsA treatment regimen. Patients were initially identified using ICD 9/10 codes if they were seen in the Cleveland Clinic Center for Functional Medicine or the Department of Rheumatology between August 2016 and January 2018. They were included into the study if they met inclusion criteria for diagnosis of RA or PsA. Statistical analysis was performed to identify any confounding variables such as age, gender, treatment regimen smoking status etc.**

**All practitioners caring for patients followed consistent treatment regimens based on professional guidelines put forth by each respective specialty.**

**It is well established that patients with RA and PsA are treated with biologic and non-biologic DMARDs in a treat to target approach outlined by several national and international guidelines.**

- **Ward MM, Deodhar A, Akl EA, Lui A, Ermann J, Gensler LS, et al. American College of Rheumatology/Spondylitis Association of America/Spondyloarthritis Research and Treatment Network 2015 recommendations for the treatment of ankylosing spondylitis and nonradiographic axial spondyloarthritis *Arthritis Rheumatol* 2016; 68: 282– 98.**
- **Singh J, Saag KG, Bridges SL Jr, Akl EA, Bannuru RR, Sullivan MC et al. Special Article: 2015 American College of Rheumatology Guideline for the treatment of Rheumatoid Arthritis *Arthritis Care Res* 2016; 68: 1– 25.**

**In the Functional Medicine department, patients were evaluated by clinicians to determine if there were areas of lifestyle (nutrition, stress, movement, sleep) that could be addressed, along with an evaluation of potential triggers that maybe exacerbating inflammation. Treatment recommendations were based on this approach, as delineated in the Institute of Functional Medicine Publication “AutoImmunity: A Guide for Functional Medicine Practitioners”.**

- **Luby R, Sennett C, Hanaway P, et al, eds. Autoimmunity: A Functional Medicine Guide. Institute for Functional Medicine; 2019.**

4. The authors use concepts without a clear definition of them. For example, alterations in intestinal permeability are discussed but it is not said how this alteration was detected. Terminology, such as biotransformation or liver detoxification strategies, is also used, without a clear definition of what this means. Note that many readers are unfamiliar with this terminology and what its measurement standards are.

**We agree with reviewers these are novel terms which can present challenges to the reader. We have clarified these less familiar terms for the readers to make these clearer:**

“The functional medicine diagnostic and treatment approach for RA and PsA focused on improving nutrition and digestive function, removing damaging environmental exposures, and providing longitudinal coaching support by way of a multi-disciplinary team of caregivers. **All patients were instructed to follow an anti-inflammatory elimination diet (specifically no processed foods, sugar, gluten and dairy) based on a majority of whole, low glycemic index foods, good fats (MUFA’s and omega 3 fats), fiber and phytonutrients. Nutrient levels were optimized and guided by laboratory results such as vitamin D or omega 3 fatty acids. If patients had altered intestinal permeability, assessed through stool tests or antibodies to lipopolysaccharide, digestive support including probiotics and prebiotics was recommended. In addition, patients with elevated levels of heavy metals such as mercury or lead (measured in whole blood or urine) would be treated to eliminate heavy metals.** Patients saw a physician or advanced practitioner, registered dietitian and health coach as part of the initial visit and afterwards for ongoing support and to make adjustments to the program based on patient response and laboratory evaluations.”

5. In the sense of the previous paragraph, you mention that the potential overexposure to heavy metals was assessed, but we are not told by what method and what values were used for this purpose.

**As previously stated in #4, we have provided more detailed information about overexposure to heavy metals:**

“The functional medicine model of care expands upon the conventional medicine model of care by addressing underlying causes, symptoms and functional imbalances associated with various biological functions. To remedy imbalances, functional medicine uses food as a first line therapy to prevent, treat and reverse chronic disease. Specifically, the diagnostic and treatment approach to RA and PsA focused on improving nutrition and digestive function, removing environmental exposures, and providing longitudinal support by way of a multi-disciplinary team of caregivers. All patients were instructed to consume an anti-inflammatory elimination diet (specifically no processed foods, sugar, gluten and dairy) based on whole, low glycemic index foods, good fats (MUFA’s and omega 3 fats), fiber and phytonutrients. Nutrient levels were optimized and guided by laboratory results such as vitamin D or omega 3 fatty acids. If patients had altered intestinal permeability, digestive support including probiotics and prebiotics was recommended. In addition, patients with elevated levels of heavy metals such as mercury or lead **(measured in whole blood or urine)** may have been treated to increase elimination. Patients saw a physician or advanced practitioner, registered dietitian and health coach as part of the initial visit and afterwards for ongoing support and to make adjustments to the program based on patient response and laboratory evaluations.”

6. Many readers will not be familiar with the PROMIS tool and its standards. Provide more information about it.

**We appreciate the PROMIS may not be familiar to some readers. A description of PROMIS has been provided in a dedicated section focusing on this point and more detailed information about the PROMIS tool was added:**

**“**Data Collection

Data was collected by retrospective chart review and was stored in a RedCap database.

Patient-Reported Outcomes

**The outcomes of the study were 12 weeks to baseline changes in pain and PROMIS global physical and mental health scores. PROMIS is a set of validated patient-centered measures, developed by the National Institute of Health, that evaluate and monitor physical, mental and social health. This patient self-administered questionnaire was created to be relevant across all conditions for assessment of functionality, symptoms, behaviors and feelings[10]. In particular, this study utilized the PROMIS Scale v1·2- Global Health measure at baseline and at 12 weeks. PROMIS is a precise and reliable way to measure domains of physical, mental and social health that are critical to PtGA.^6^** Pain scores were collected using visual analog scale ranging from 0-10 at baseline and 12 weeks.”

7. Some of the statistical tests used as the ANOVA are based on assuming normality in the distribution of the quantitative variables. In general, this test seems more appropriate to compare the means of 3 or more normal variables. The hypothesis of normality in the distribution of the study variables was tested?

**Thank you for pointing this out.**

**ANOVA is generally robust to small departures from normality assumptions.  We examined our distributions graphically and found no gross evidence of non-normality.  In addition, nonparametric analyses were run as a sensitivity analysis and provided similar results, and are not presented.**

**ANOVA is not strictly restricted to compare the means of 3 or more groups. It can be applied to any number of groups. When there are only 2 groups to be compared, its result is equal to two-sample t-test. To avoid confusion, we have changed the table footnote to two-sample t-test, whenever this test was used.**

8. To define how relevant this type of intervention can be, it would be useful to provide an effect size of the differences found, not only if these differences were significant or not. Only in this way can we know the magnitude of this intervention on the general health of these patients.

**We agree with the reviewer that it would be beneficial to include effect sizes to help readers understand the relevance of the intervention. We have now included the effects size in Table 2:**

|  | **Overall**  **(N=109)** | | **Functional Medicine**  **(N=54)** | | **Standard of care alone**  **(N=55)** | |  |  |
| --- | --- | --- | --- | --- | --- | --- | --- | --- |
| **Factor** | **N** | **Statistics** | **N** | **Statistics** | **N** | **Statistics** | **Cohen's d** | **p-value** |
| Global physical health T  score at 12 weeks | 109 | 42.8 ± 7.3 | 54 | 45.4 ± 6.5 | 55 | 40.2 ± 7.1 | **0.76** | ***<0.001^a1^*** |
| Global mental health T-score  at 12 weeks | 109 | 46.5 ± 8.5 | 54 | 46.8 ± 8.9 | 55 | 46.1 ± 8.2 | **0.08** | 0.70^a1^ |
| Pain at 12 weeks | 108 | 4.1 ± 2.3 | 54 | 3.1 ± 1.8 | 54 | 5.0 ± 2.4 | **-0.90** | ***<0.001^a2^*** |
| RAPID 3 at 12 weeks | 52 | 3.8 ± 2.0 | 6 | 2.1 ± 0.78 | 46 | 4.0 ± 2.0 | **-0.99** | ***<0.001^a2^*** |
| Physical Health Score  Change | 109 | 1.3 ± 5.0 | 54 | 2.2 ± 4.9 | 55 | 0.48 ± 5.0 | **0.35** | 0.068^a1^ |
| Mental Health Score Change | 109 | 0.90 ± 4.7 | 54 | 0.94 ± 4.8 | 55 | 0.86 ± 4.7 | **0.02** | 0.93^a1^ |
| Pain Score Change | 108 | -0.31 ± 1.9 | 54 | -0.41 ± 1.6 | 54 | -0.20 ± 2.1 | **-0.11** | 0.57^a1^ |
| Statistics presented as Mean ± SD.  p-values: a1=t-test, a2=Satterthwaite t-test. | | |  |  |  |  |  |  |

**The following was also added the manuscript text:**

“On univariate analysis, 12 week PROMIS global physical health T scores (45·4 ±6·5 and 40·2 ± 7·1) and pain scores (3·1 ± 1·8 and 5·0 ± 2·4) remained statistically significant between the functional medicine and standard of care groups respectively, however, there was no statistically significant change when comparing groups to each other. **In terms of the effect sizes (Cohen’s d), at univariate level, 12-week pain scores and RAPID 3 had biggest effect sizes of around 0.9, suggesting that the mean differences of these two measures equal to around 0.9 of standard deviation (Table 2). “**

General comment:
As the authors point out, the main and most serious problem of the study is its retrospective design, which generates many uncertainties regarding the reliability of the results. On the other hand, the number of patients is small, and the authors chose two very different types of patients. It is known that, for example, metabolic syndrome, a factor that has a very negative impact on the health of these patients, is much more common in psoriatic than in rheumatoid arthritis, therefore, both entities must be approached differently. In fact, there are already experiences in the literature on the benefits of weight loss on the outcomes reported in PsA. Therefore, it is advisable that the authors undertake a prospective randomized clinical trial to give a clear role to functional medicine in the management of these diseases.

Reviewer #3: The authors conduct a 12-week, retrospective study to investigate the impact of functional medicine on patient reported outcomes in patients with inflammatory arthritis. The data were collected at baseline and 12 weeks based on 54 subjects. The results showed that the reduction in pain, change in PROMIS physical health score and changes in global mental health scores were significantly different between the functional medicine group and the standard of care.

1. Line 102. It’s unclear how the study subjects were recruited. Sampling approach? Recruitment period? It lacks of details.

**We appreciate concern regarding recruitment bias. In our study, we looked at all** p**atients with an ICD 9/10 code corresponding to rheumatoid arthritis or psoriatic arthritis over a period from August 2016-January 2018. Inclusion and exclusion criteria were strictly followed for all patients. This was clarified in the manuscript:**

**“**In this retrospective, single center study, we evaluated the change in Patient Reported Outcomes Measurement Information System (PROMIS®) global physical health, PROMIS global mental health, and pain scores in patients who underwent a functional medicine program (n = 54) adjunctive to the standard of care as compared to patients who received standard of care alone (n = 55). The study protocol was reviewed and approved by the Institutional Review Board at the Cleveland Clinic Foundation (IRB#17-237). **Patients aged 18 and older who were seen as a new patient in the Cleveland Clinic Center for Functional Medicine between August 2016 and January 2018 were screened for inclusion into the study by diagnosis code for RA(ICD 9: 714·0; ICD 10: M06·9) or PsA(ICD 9: 696·0; ICD10: L40·50). Patients were eligible for inclusion if they had documentation by a board certified rheumatologist at our institution confirming their diagnosis of RA or PsA and were on stable doses of prednisone and DMARD therapy throughout the study period. Patients were compared to controls seen only in the Rheumatology Department during the same time period. Patients in the standard of care group were matched to the functional medicine group based on similarity in baseline characteristics, such as age, sex, smoking status, and disease seropositivity, if applicable.”**

2. Line 144. Typo, “rheumatoid factorwere summarized…”. Should be “factor were”?

**This was changed to factor were.**

3. Line 145. “to test difference in continuous variables”. It’s unclear what difference are discussing here.

**Continuous variables identified included age and patient reported outcomes. This was clarified in the text:**

Summary statistics were calculated using means and standard deviations for continuous variables, such as age and patient reported outcomes. The normality of the continuous variables was tested using Shapiro-Wilk tests. Two-sample t-tests were used to compare the differences in continuous variables that meet the normality assumption, **such as Global Physical and Mental Health T-scores at 12 weeks and the 12-week to baseline change in Global Physical Health T-score**. Categorical variables, such as sex, smoking status anti-cyclic citrullinated peptide, rheumatoid factor were summarized using frequencies and percentages and were compared using Pearson’s chi-square tests. To obtain the effect sizes, Cohen’s ds were provided for patient reported outcomes at 12 weeks and the changes in scores.

4. Line 163. “54 patients seen in the rheumatology department alone during the same time period….” It’s unclear which group it refers to. Based on the numbers, it seems refer to the group with functional medicine; however, if so, how the group receiving the standard of care alone was recruited?

**Thank you for the feedback. We realize the way this was written was confusing for readers and have changed it in the manuscript to:**

**“318 patients seen in the functional medicine department were initially identified as having RA or PsA by ICD 9/10 code. Of these patients, 54 had documentation of their diagnosis by a board certified rheumatologist and had complete data over the study time frame and were included into the study.** The main reasons for exclusion from the study were: incomplete data, inadequate follow up within the first 12 weeks after starting the functional medicine program, inadequate documentation of rheumatologic diagnosis or confounding by lack of stable doses of medications to treat inflammatory arthritis. In the standard of care group, 54 patients seen in the rheumatology department alone without any functional medicine intervention were identified for inclusion into the study. Baseline characteristics were similar between both groups with the exception of PROMIS global physical health and pain scores which were significantly different in the functional medicine group as compared to the standard of care group (PROMIS global physical health score 43·2 ± 6·6 in the functional medicine group as compared to 39·7 ± 8·7 in the standard of care group and pain scores of 3·5 ± 1·9 and 5·2 ± 2·7 respectively) (Table 1).”

5. Table 1. Two of three primary factors (physical health, pain score) were significantly different at baseline between two groups. Was the type of assigned care determined based on those factors during the recruitment?

**We appreciate that there were statistically significant differences between some baseline characteristics. In order to address this, we used a multivariable analysis to account for these differences. Using this model we were able to demonstrate a statistically significant improvement in primary outcomes.**

**In this retrospective study, patients were not assigned to care. Each group was defined using the exclusion and inclusion criteria and were not assigned to a group.**

6. PLOS authors have the option to publish the peer review history of their article ([what does this mean?](https://protect-us.mimecast.com/s/Ju8DCpYKxWSnAlDGkivrA_g?domain=journals.plos.org)). If published, this will include your full peer review and any attached files.


If you choose “no”, your identity will remain anonymous but your review may still be made public.


**Do you want your identity to be public for this peer review?** For information about this choice, including consent withdrawal, please see our [Privacy Policy](https://protect-us.mimecast.com/s/jZz5Cqx5yWS8X4WDBhYj_xL?domain=plos.org).

Reviewer #1: **Yes:** Alex Vasquez

Reviewer #2: No

Reviewer #3: No

[NOTE: If reviewer comments were submitted as an attachment file, they will be attached to this email and accessible via the submission site. Please log into your account, locate the manuscript record, and check for the action link "View Attachments". If this link does not appear, there are no attachment files.]

While revising your submission, please upload your figure files to the Preflight Analysis and Conversion Engine (PACE) digital diagnostic tool, [https://pacev2.apexcovantage.com/](https://protect-us.mimecast.com/s/6ZdWCrk5zWt82N9pMh69D08?domain=pacev2.apexcovantage.com). PACE helps ensure that figures meet PLOS requirements. To use PACE, you must first register as a user. Registration is free. Then, login and navigate to the UPLOAD tab, where you will find detailed instructions on how to use the tool. If you encounter any issues or have any questions when using PACE, please email PLOS at [figures@plos.org](mailto:figures@plos.org). Please note that Supporting Information files do not need this step.

*In compliance with data protection regulations, you may request that we remove your personal registration details at any time.* [*(Remove my information/details)*](https://protect-us.mimecast.com/s/Swz3Cv25E6U7ABXG1HYHVqm?domain=editorialmanager.com)*. Please contact the publication office if you have any questions.*
